# Teamwork, communication and exchange despite Covid-19 – experiences from a digital elective in human medicine studies as part of the HiGHmed project

**DOI:** 10.3205/zma001379

**Published:** 2020-12-03

**Authors:** Marianne Behrends, Ina Hoffmann, Michael Marschollek, HiGHmeducation Consortium

**Affiliations:** 1 Hannover Medical School, Peter L. Reichertz Institute for Medical Informatics, Hannover, Germany; 2 HiGHmed Working Group for Teaching and Training, Heidelberg, Germany

**Keywords:** digital teaching, digitization of medicine, data literacy, medical studies, medical informatics

## Abstract

**Introduction:** In order to promote training and further education on topics related to the digitization of medicine, the *HiGHmeducation* consortium is developing online learning modules. These modules could also be offered across locations. For students of human medicine, an elective for the acquisition of data literacy has been implemented. Originally designed as a blended learning offer, the elective was then carried out completely online due to the Covid-19 pandemic. Despite the lack of classroom teaching, the aim was to achieve intensive cooperation between the students.

**Project description:** In the elective, the students worked on a total of 14 learning tasks, so-called *e-tivities*, which stimulate collaborative work and thus promote the examination of the learning content. These asynchronous learning activities were supplemented by video conferences, in which the students also took on the role of presenters. The teachers accompanied this learning process as e-moderators.

**Results: **In April/May 2020, the elective course was carried out with 12 students entirely online. Despite a workload that was experienced as high, the elective was rated very well by the students.

**Discussion: **The didactic concept of the elective enabled an active engagement with the learning material and the social interaction between the learners. With the digital learning offers, the learners were able to gain new experiences which are also of professional relevance.

**Conclusion: **The didactic concept of the elective can be transferred to other courses. Future studies must show which long-term learning effects can be generated by digital teaching based on teamwork, communication and exchange.

## Zusammenfassung

**Einleitung: **Um die Aus- und Weiterbildung zu Themen rund um die Digitalisierung der Medizin zu fördern, werden im Verbund *HiGHmeducation* Online-Lernmodule entwickelt, die auch standortübergreifend angeboten werden können. Für das Humanmedizinstudium wurde ein Wahlfach II für den Erwerb von Datenkompetenzen umgesetzt, das ursprünglich als Blended Learning Angebot konzipiert, während der Covid-19-Pandemie dann vollständig online durchgeführt wurde. Trotz fehlender Präsenzlehre sollte dabei eine intensive Zusammenarbeit der Studierenden erreicht werden.

**Projektbeschreibung:** Im Wahlfach bearbeiteten die Studierenden insgesamt 14 Lernaufgaben, sogenannte *E-tivities*, die eine kollaborative Zusammenarbeit anregen und damit die Auseinandersetzung mit den Lerninhalten fördern. Diese asynchronen Lernaktivitäten wurden durch Videokonferenzen ergänzt, bei denen die Studierenden auch die Rolle der Präsentierenden übernahmen. Die Lehrenden begleiteten als E-Moderator_innen diesen Lernprozess.

**Ergebnisse: **Im April/Mai 2020 wurde das Wahlfach mit 12 Studierenden vollständig online durchgeführt. Trotz einer als hoch erlebten Arbeitsbelastung wurde das Wahlfach von den Studierenden sehr gut bewertet.

**Diskussion:** Eine aktive Auseinandersetzung mit dem Lernstoff und die soziale Interaktion zwischen den Lernenden konnte mit dem didaktischen Konzept des Wahlfachs erreicht werden. Durch das digitale Lernangebot konnten die Lernenden neue Erfahrungen sammeln, die auch von beruflicher Relevanz sind.

**Schlussfolgerung:** Das didaktische Konzept des Wahlfachs lässt sich auf andere Lehrveranstaltungen übertragen.

Zukünftige Untersuchungen müssen zeigen, welche längerfristigen Lerneffekte eine digitale Lehre, die auf Teamarbeit, Kommunikation und Austausch setzt, generieren kann.

## 1. Introduction

As part of the Medical Informatics Initiative of the Federal Ministry of Research and Education [https://www.medizininformatik-initiative.de/], the *HiGHmed* project [https://www.highmed.org/] aims at contributing to the improvement of medical research and patient care through IT solutions. In order to simultaneously promote education and training in this area, *HiGHmeducation* [https://education.highmed.org/], an association of universities and an industrial partner, was constituted. Its goal is to develop online learning modules that can also be offered across locations.

Especially for the integration into the study of human medicine, an elective on data literacy [1] has been developed. The students of this elective acquire the skills needed to collect, manage, evaluate and apply data in a critical manner through a variety of learning activities. An independent and scientifically reflected examination of the topic within a learning community [2] is enabled by the central didactic approach.

After the first implementation of the elective at the beginning of 2020 as a blended learning offer with online learning phases and regular face-to-face meetings, the second implementation during the Covid-19 pandemic took place entirely online. Despite the lack of face-to-face teaching, an intensive exchange between the students should be achieved.

## 2. Project description

The design of the elective is based on the didactic concept of *HiGHmed* [3] and the model of *e-tivities* of Salmon [4], which follow a constructivist approach and focus on cooperative learning. Work tasks in the form of e-tivities stimulate independent work and exchange between the learners. Starting with an activating introduction to the topic, the learners first work on a concrete task on their own. They present their results to the other learners in a forum. In the next step, each *e-tivity* contains a work assignment that requires cooperation and thus trains competencies such as teamwork and communication skills. The final outcome of each *e-tivity* is a written elaboration of the common result. As e-moderators, the teachers accompany this learning process by providing thought-provoking impulses, linking knowledge, giving feedback and encouraging cooperation. 

For collaborative work, students use forums and wikis in the university's learning management system. In the case of the pure online implementation, they were also given the opportunity to meet in a virtual room in the form of a video conference. The three face-to-face sessions of the first implementation were replaced by four video conferences, in which the students also took on the role of presenters to show the results of their work on the e-tivities. 

In addition to *e-tivities* for getting to know each other, for getting started and for reflection, the topics “data collection and sources of medical data”, “data management and modelling”, “data analysis and visualisation” as well as “ethical and legal aspects” were dealt with. In total, the students had to work on 14 *e-tivities*.

## 3. Results

In April/May 2020, the elective course was carried out completely online with 12 students over a period of five weeks. As with all *HiGHmed* modules, the social exchange, the support by the teachers, and the possibility of joint knowledge construction through communication were examined based on the German translation of the *Community of Inquiry* questionnaire [5], [6]. All aspects were positively evaluated by the students on average. As part of the regular teaching evaluation of the university, the students rated the elective course as very positive (13.1 points on a scale between 0 (worst) and 15 (best)). Other aspects examined in the evaluation also show the weaknesses of the didactic approach. For example, the workload and asynchronous exchange in the forums were experienced as a challenge by some students. Nevertheless, the positive feedback was predominant and the intensity of the exchange was experienced by the students – as well as by the teachers – as enriching. In the free texts, the clear structure of the tasks, the discussions in the forums, the video conferences and the opportunity to work independently on the topics in the team were highlighted as positive examples of digital teaching.

## 4. Discussion

The active examination of the learning material and the social interaction between the learners are challenges that influence learning success, especially in pure digital online courses [7]. The form of the elective course, which is based on constructivist learning theories, offered the students appropriate support, but also required motivation and work effort. The students experienced this new form of learning as an enrichment. For example, they were able to gain initial experience with an active role in video conferences, which can form a basis for the implementation of telemedicine like online consultation hours, the importance of which has increased significantly with the Covid-19 pandemic [https://www.management-krankenhaus.de/topstories/it-kommunikation/corona-krise-beschleunigt-telemedizin].

## 5. Conclusion

The teaching restrictions caused by Covid-19 open opportunities for new learning experiences for students and teachers alike. The presented didactic concept can be transferred to other courses with smaller learning groups.

With regard to the digitization of medicine, the cooperation between the disciplines of medicine and computer science plays an important role. In order to promote this exchange, *HiGHmeducation* offers digital learning modules across disciplines and universities. Further research will have to show which long-term learning-effects the chosen approach of digital teaching can generate as part of an interdisciplinary education in medical studies, relying on teamwork, communication and exchange.

## Competing interests

The authors declare that they have no competing interests. 
